# Preparation and Multifunctional Properties of Hydrophobic Electrospun Cellulose Acetate/Polystyrene Nanofibrous Membranes

**DOI:** 10.3390/ma19184000

**Published:** 2026-09-20

**Authors:** Zijia Wang, Fujuan Liu

**Affiliations:** National Engineering Laboratory for Modern Silk, College of Textile and Clothing Engineering, Soochow University, 199 Ren-Ai Road, Suzhou 215123, China; 20245215006@stu.suda.edu.cn

**Keywords:** electrospinning, polystyrene, cellulose acetate, hydrophobic nanofibrous membranes

## Abstract

In this work, cellulose acetate (CA)/polystyrene (PS) composite nanofibrous membranes with various mass ratios (0/1, 1/1, 3/1, 5/1, 11/1, and 23/1) were fabricated via one-step electrospinning. The effects of the CA/PS ratio on fiber morphology, mechanical properties, porosity, wettability, air permeability, and thermal insulation were systematically investigated. Compared with the pure PS nanofibrous membranes, the CA/PS (3/1) membrane shows substantially enhanced structural and functional properties: porosity increases from 27.38% to 53.27%, air permeability from 56.45 ± 3.71 mm/s to 104.51 ± 2.89 mm/s, and water contact angle (WCA) from 124.79 ± 3.21° to 132.09 ± 0.86°. Its tensile strength (5.38 ± 0.47 MPa) and elongation at break (47.12 ± 3.28%) are approximately 3.6 and 6.1 times those of the pure PS membrane (1.50 ± 0.27 MPa and 7.67 ± 1.61%). These prominent multifunctional characteristics enable the CA/PS composite nanofibrous membranes to serve as promising candidates for personal protection, filtration and separation, and thermal management applications, although application-specific performance and long-term durability still require further investigations.

## 1. Introduction

Hydrophobic nanofibrous porous membranes have attracted extensive attention in separation and protective applications due to their unique structural features and tunable wettability [1,2,3]. Electrospinning is a versatile, scalable, and efficient technique for nanofiber fabrication, enabling the continuous production of micro/nanoscale fibers from polymer solutions or melts. The electrospun membranes possess high specific surface area, abundant porosity, and interconnected pore networks [4,5], rendering them promising for diverse advanced applications, including filtration and separation [6], tissue engineering [7], sensing and detection [8], and personal protective equipment [9]. Furthermore, the electrospinning process offers considerable tunability, allowing precise modulation of fiber morphology, microstructure, and surface chemistry through parameter optimization and post-treatment strategies [10]. However, single-component electrospun nanofibrous membranes generally suffer from poor mechanical properties, which severely limits their practical applications [11]. To address this bottleneck, numerous efforts have been dedicated to enhancing the mechanical performance of fibrous membranes through various methods, such as filler incorporation [12,13], spinning parameter optimization [14], post-treatment modification [15,16,17], and structural design [18,19]. Among these approaches, polymer blending has emerged as a simple, low-cost, and effective strategy for membrane performance optimization [20,21,22].

Polystyrene (PS) is a widely used thermoplastic polymer with low surface energy due to the abundant hydrocarbon groups on its molecular chains, which endows PS with excellent hydrophobic and lipophilic characteristics. PS also offers favorable chemical stability, low cost, and good processability [23]. Owing to its superior spinnability, PS serves as an ideal matrix for fabricating hydrophobic fibrous membranes. Nevertheless, the inherent brittleness of pure PS severely undermines its structural stability and long-term service durability, particularly in oil–water separation applications [24]. To improve the mechanical properties of PS fibrous membranes, various polymers have been blended with PS for electrospinning, including thermoplastic polyurethane (PU) [25], polyacrylonitrile (PAN) [26], polyvinylidene fluoride (PVDF) [27], and polyamide 6 (PA6) [28]. Despite the enhanced mechanical strength achieved through these blending systems, most modified membranes tend to compromise functional properties, such as reduced adsorption capacity [29] or deteriorated air permeability, making it challenging to obtain well-balanced properties for practical applications.

Cellulose acetate (CA) refers to a widely available, water-insoluble, and biodegradable cellulose derivative, consisting of β-D-glucose units linked by β(1→4) glycosidic bonds. CA can be processed into highly porous fibrous membranes via electrospinning, showing great potential for various applications [30]. Blending CA with PS holds promise for maintaining the hydrophobic nature of PS while modulating fiber morphology and pore-channel structures through structural variations originating from the thermodynamic incompatibility between the two polymers. However, pure CA suffers from poor electrospinnability in single-solvent systems [31]. Continuous and uniform fibers are difficult to obtain, and are accompanied by frequent beading and fiber-breakage defects. Therefore, blending modification is generally adopted to enhance its electrospinnability.

Several studies have reported the preparation of CA/PS blend nanofibers. Rosdi et al. [32] prepared electrospun CA/PS blend nanofibers with mass ratios ranging from 20:80 to 80:20, yet their investigation was primarily limited to thermal properties and surface morphology. Abosedira et al. [33] fabricated CA/PS electrospun fibers for atmospheric water harvesting, reporting a water contact angle of 124.7° and a water collection rate of 61.9 mg/cm^2^·h for the 1:2 blend; however, mechanical properties, porosity, air permeability, and thermal insulation were not addressed. In contrast to these earlier studies, the present work extends the blend composition toward much higher CA loading (up to CA/PS = 23/1) and systematically investigates the effects of CA/PS mass ratio on fiber morphology, chemical composition, thermal stability, mechanical performance, air permeability, and hydrophobicity. This study aims to develop multifunctional nanofibrous membranes with integrated merits of improved mechanical strength, high porosity, outstanding air permeability, and hydrophobicity. The outcomes are expected to offer feasible material candidates and fundamental references for the design and preparation of advanced membranes in oil–water separation, air filtration, as well as thermal insulation applications.

## 2. Experimental

### 2.1. Materials

Cellulose acetate (CA, M_n_ = 53,000 g/mol) was obtained from Shanghai Macklin Biochemical Technology Co., Ltd. (Shanghai, China); Polystyrene (PS, general grade III, M_w_ = 10,414 g/mol) was purchased from Shanghai Aladdin Biochemical Technology Co., Ltd. (Shanghai, China). N,N-Dimethylformamide (DMF), tetrahydrofuran (THF), and n-butanol were all provided by Jiangsu Qiangsheng Functional Chemistry Co., Ltd. (Changshu, Jiangsu, China). Methyl Orange (MO) was obtained from Shanghai Sinopharm Chemical Reagent Co., Ltd. (Shanghai, China), and Methylene Blue (MB) was from Shanghai Qiangshun Chemical Co., Ltd. (Shanghai, China). Deionised water was self-prepared in the laboratory. All reagents were analytically pure.

### 2.2. Preparation of CA/PS Nanofibrous Membranes

N, N-Dimethylformamide (DMF) and tetrahydrofuran (THF) were mixed at a mass ratio of 2:1 to prepare the spinning solvent. Cellulose acetate (CA) and polystyrene (PS) were dissolved in the mixed solvent at varied CA/PS mass ratios of 0/1, 1/1, 3/1, 5/1, 11/1, and 23/1. These ratios were selected to cover a wide composition range from pure PS to CA-dominant blends, enabling systematic investigation of composition-dependent fiber morphology and membrane properties. The total polymer concentration was fixed at 14 wt% relative to the total mass of the spinning solution. The mixtures were magnetically stirred at 400 rpm at room temperature until completely dissolved. The well-homogenized polymer solutions were then electrospun under optimized operational parameters (detailed electrospinning parameters are provided in Table S1), which were kept constant for all CA/PS blend ratios. The as-spun nanofibrous membranes (200 mm × 314 mm) were subsequently dried at 60 °C in a conventional oven for 6 h and stored in a dry environment for further characterization.

### 2.3. Characterization

#### 2.3.1. Morphological Characterization

The surface morphology of the nanofibrous membranes was observed via a cold field emission scanning electron microscope (Hitachi S-4800, Hitachi Ltd., Tokyo, Japan). SEM images were captured at magnifications of 3000× and 35,000× under an accelerating voltage of 3 kV. Fiber diameters were quantified with ImageJ software (version 1.53, National Institutes of Health, Bethesda, MD, USA). Measurements were performed on two independently prepared membrane samples, with 100 randomly selected fibers measured per sample. Diameter distribution histograms were fitted with a normal (Gaussian) distribution model using Origin 2026 (OriginLab Corporation, Northampton, MA, USA).

#### 2.3.2. FTIR Spectroscopy

The chemical functional groups and molecular structures of the fibrous membranes were examined using a Fourier transform infrared spectrometer (FTIR, NICOLET iS5, Thermo Fisher Scientific, Madison, WI, USA) in attenuated total reflectance (ATR) mode equipped with a diamond crystal. Spectra were recorded over the wavenumber range of 4000–500 cm^−1^ at a resolution of 4 cm^−1^, with 32 accumulated scans.

#### 2.3.3. Thermogravimetric Analysis

Thermogravimetric (TG) analysis (Diamond 5700, PerkinElmer, Waltham, MA, USA) was performed under a nitrogen atmosphere. Approximately 6 ± 0.5 mg of each sample was loaded into a 100 μL alumina crucible and heated from 30 to 700 °C at a heating rate of 20 °C/min. The purge gas flow rates were 40 mL/min for the balance compartment and 60 mL/min for the sample chamber. Measurements were conducted on a single representative sample per composition, as this test mainly aimed to determine the general thermal degradation behavior across the CA/PS blend system.

#### 2.3.4. Tensile Properties

The tensile properties of fibrous membranes were measured using a double-arm universal materials testing machine (Instron 3365, Instron Corporation, Norwood, MA, USA) equipped with a 10 N load cell. The specimens were cut into rectangular strips of 1 cm (width) × 4 cm (length). For each ratio, five samples were analyzed. The tests were conducted at a gauge length of 20 mm, a pre-load of 0.2 cN, and a stretching rate of 20 mm/min. The thickness of each sample was determined with a digital micrometer. The tensile stress (σ) and strain (ε) were calculated according to the following equations:

(1) $$\sigma \;=\;\frac{F}{W\times T}$$

(2) $$\varepsilon =\frac{△L}{L_{0}}$$

where F is the applied load (N), W is the specimen width (mm), T is the specimen thickness (mm), ΔL is the crosshead displacement (mm), and L_0_ is the initial gauge length (mm).

#### 2.3.5. Porosity Measurement

The porosity of the nanofibrous membranes was determined by the liquid adsorption method with n-butanol employed as the wetting liquid [34]. Two independently prepared membranes were tested for each composition, and three measurements were performed on each membrane. The membranes were cut into square pieces (2 cm × 2 cm). The thickness was measured at three randomly selected locations using a micrometer and averaged. The specimens were then immersed in n-butanol for 12 h to guarantee complete penetration of the liquid into the interconnected pores. After immersion, excess liquid on the membrane surface was gently removed with filter paper, and the wet mass was recorded. The porosity (P) was calculated as follows:

(3) $$P\;=\;\frac{\frac{M_{1}}{\rho_{1}}}{\frac{M_{1}}{\rho_{1}}+\frac{M_{0}}{\rho_{0}}}\times 100\%$$

where M_1_ and M_0_ represent the mass of n-butanol absorbed by the membrane and the mass of the pristine dry membrane, respectively, and ρ_1_ and ρ_0_ denote the densities of n-butanol and the membrane, respectively.

#### 2.3.6. Surface Wettability

Surface wettability was evaluated by recording water contact angles (WCA) using a contact angle tester (OCA40, DataPhysics Instruments GmbH, Filderstadt, Germany) with 3 μL water droplets. Five samples for each ratio were analyzed. All measurements were conducted at room temperature and 45 ± 5% relative humidity. The WCAs were recorded 10 s after droplet deposition.

#### 2.3.7. Air Permeability

The air permeability was measured using a YG461G automatic air permeability tester (Wenzhou Fangyuan Instrument Co., Ltd., Wenzhou, Zhejiang, China) at a test area of 20 cm^2^ and a differential pressure of 200 Pa. Two independent samples were tested for each composition, and three measurements were taken on each sample.

#### 2.3.8. Water Vapor Transmission Rate

To characterize the moisture permeability of the CA/PS nanofibrous membranes, the water vapor transmission rate (WVTR) was determined following a gravimetric method adapted from ASTM E96/E96M-16 (Water Method) [35]. Briefly, test tubes filled with 50 mL of deionized water were placed in an incubator at 37 °C and 50% relative humidity for 12 h. Two independently prepared samples were evaluated for each composition, and three measurements were performed on each sample. The total mass loss was recorded upon test completion (ΔW_12h_). The formula for WVTR calculation is shown below:

(4) $$\mathrm{WVTR}=\frac{W_{loss}}{S}$$

where W_loss_ is defined as the daily mass loss of water (kg/day), calculated by doubling the 12 h mass loss (i.e., W_loss_=ΔW_12h_ × 2) and S (m^2^) stands for the open area of the test tube.

#### 2.3.9. Thermal Insulation Performance

The surface temperature distribution and thermal insulation performance of the CA/PS nanofibrous membranes were analyzed with an infrared thermal imager (FOTRIC, Shanghai FOTRIC Thermal Imaging Technology Co., Ltd., Shanghai, China).

#### 2.3.10. Statistical Analysis

All data were expressed as mean ± standard deviation (SD). Statistical analysis was performed using SPSS software (version 27.0, IBM Corp., Armonk, NY, USA). One-way analysis of variance (ANOVA) followed by Tukey’s honest significant difference (HSD) post hoc test was applied for multiple pairwise comparisons. Groups marked with different letters are significantly different (*p* < 0.05), whereas groups sharing identical letters show no significant difference (*p* > 0.05).

## 3. Results and Discussion

### 3.1. Morphology of Nanofibrous Membranes

**Figure 1:**
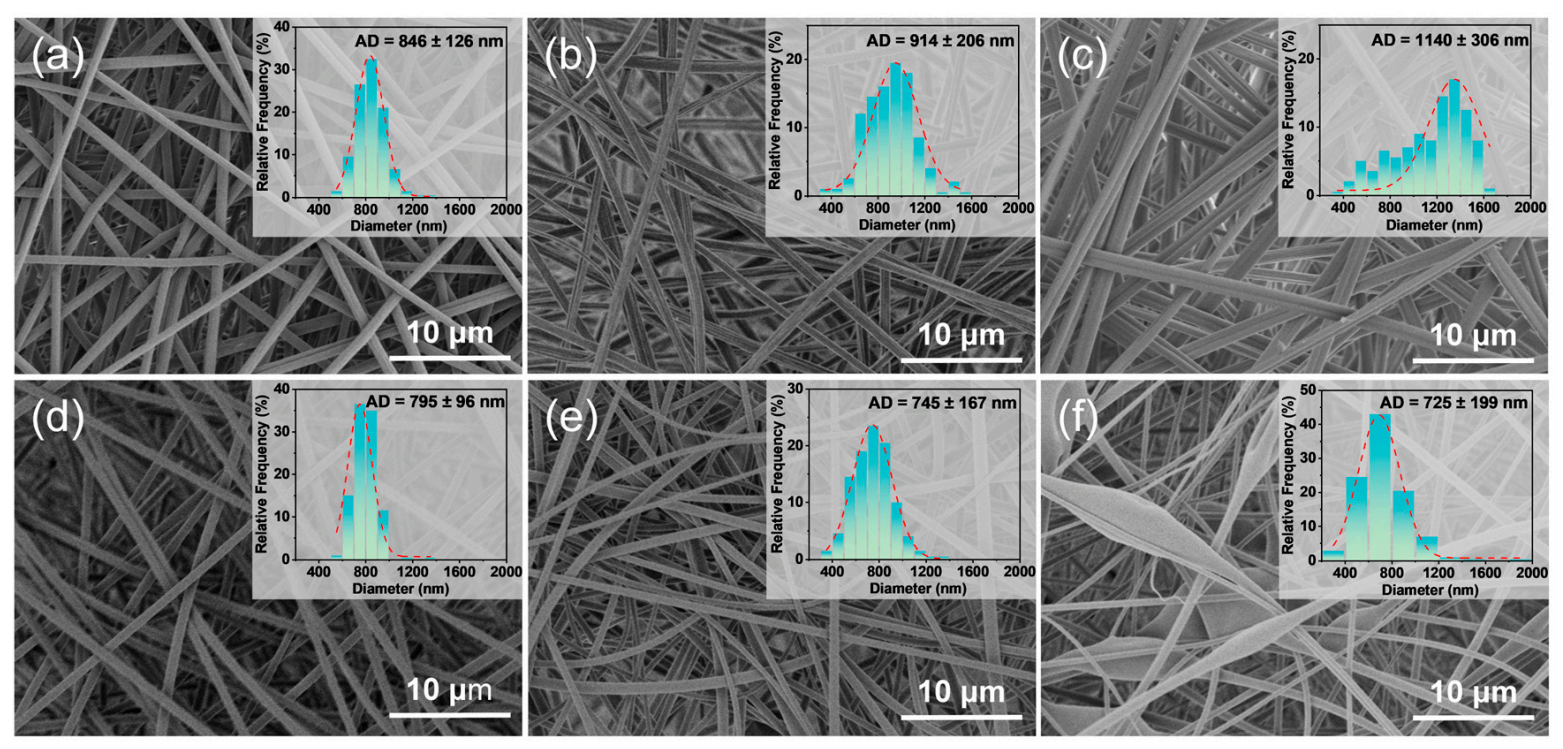
Morphology and diameter distribution of electrospun CA/PS nanofibers at various CA/PS mass ratios: (**a**) 0/1, (**b**) 1/1, (**c**) 3/1, (**d**) 5/1, (**e**) 11/1, (**f**) 23/1. Data are expressed as the mean ± SD (n = 200). The red dashed lines indicate the normal (Gaussian) distribution fitting curves.

Figure 1 presents the SEM images and corresponding fiber diameter distributions of electrospun CA/PS nanofibrous membranes with diverse blending ratios. The pure PS membrane (CA/PS = 0/1) exhibits uniform fibrous structures with an average fiber diameter of 846 ± 126 nm. As the CA content increases to CA/PS = 3/1, the average fiber diameter increases to 1140 ± 306 nm with a broader distribution. This enlargement in fiber diameter originates from the significant rise in solution viscosity, which increases from 273.67 ± 9.07 mPa·s (pure PS) to 2618.67 ± 25.66 mPa·s (CA/PS = 3/1) (Figure S1a). Such elevated viscosity enhances polymer chain entanglements, which in turn hinders the stretching of the ejected jet during electrospinning. However, when the viscosity further increased to 5842.80 ± 67.80 mPa·s (CA/PS = 23/1), prominent bead-on-string defects appeared on the fiber surface. Pure CA could not be electrospun into continuous fibers under the same conditions in this study (Figure S2). This phenomenon can be tentatively attributed to the high surface tension resulting from the strong intermolecular interactions among CA chains [36].

**Figure 2:**
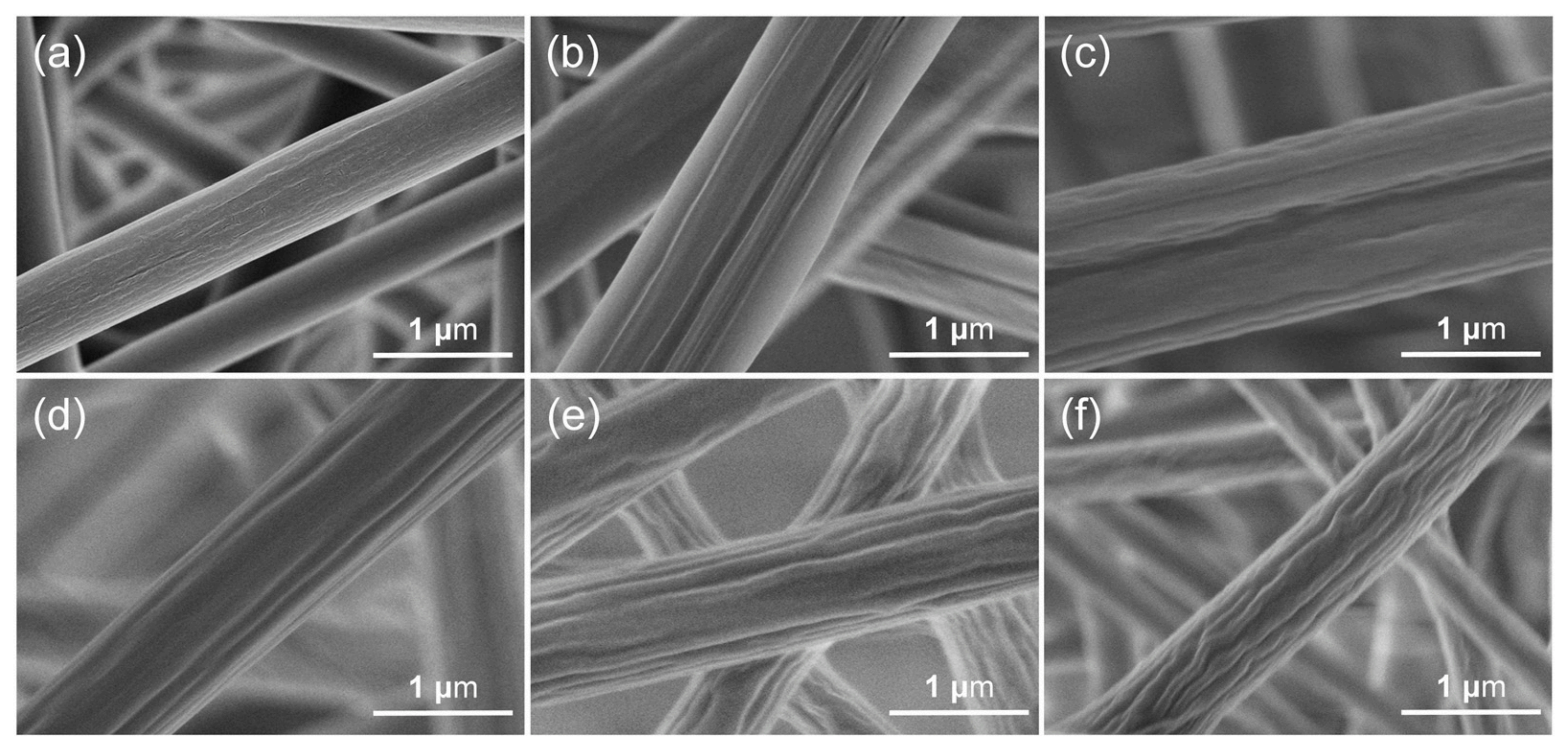
High-magnification SEM images of electrospun CA/PS nanofibers at various CA/PS mass ratios: (**a**) 0/1, (**b**) 1/1, (**c**) 3/1, (**d**) 5/1, (**e**) 11/1, (**f**) 23/1.

Figure 2 presents high-magnification SEM images of CA/PS membranes with various blending ratios. The pure PS fibers (Figure 2a) exhibit a relatively smooth surface with some fine cracks, which can be attributed to the rapid evaporation of THF from the jet surface. During electrospinning, THF (boiling point 66 °C) evaporates much faster than DMF (boiling point 153 °C) [37]. This rapid evaporation of THF enriches the jet surface in polymer and promotes the formation of a semi-solid shell, while the jet core remains DMF-rich and fluid [38]. With increasing CA content, distinct longitudinal grooves and wrinkled structures gradually emerge on the fiber surface. The emergence of this unique texture can be ascribed to the combined effects of solvent evaporation and phase separation between CA and PS. CA and PS are thermodynamically incompatible [39], which may lead to macroscopic phase separation in the solution state, potentially generating CA-rich and PS-rich domains, which is possibly reflected by the milky appearance of the mixed solution (Figure S3). These domains may persist during solidification, and their differential shrinkage, together with core shrinkage, can potentially induce buckling instabilities [40], forming wrinkles that evolve into longitudinal grooves under the electric field [41]. 

### 3.2. FTIR Spectrum Analysis

**Figure 3:**
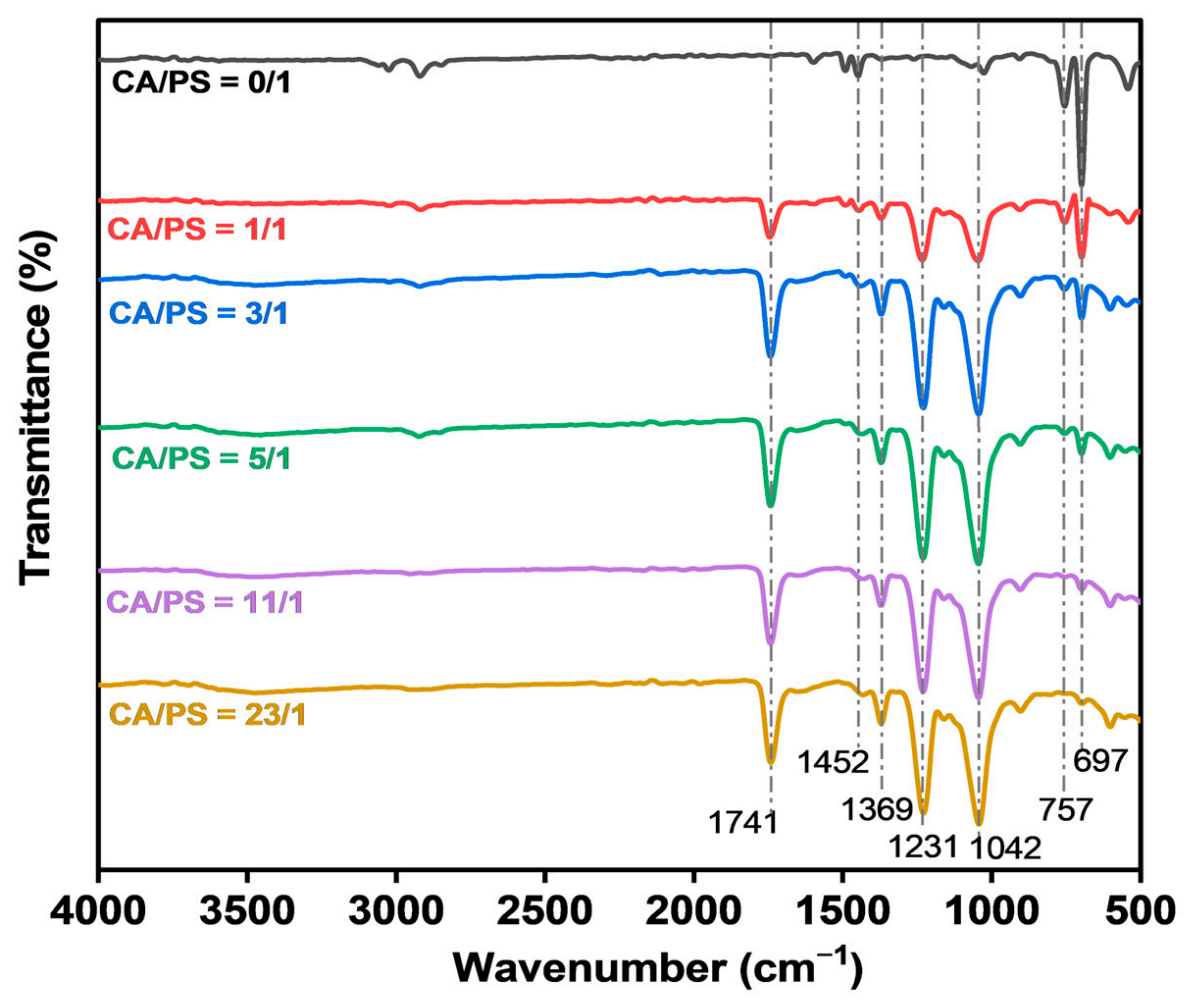
FTIR spectra of CA and CA/PS membranes with different CA/PS ratios.

The FTIR spectra of electrospun CA/PS nanofibrous membranes with various blending ratios are shown in Figure 3. The characteristic absorption peaks of the pure PS membrane (CA/PS = 0/1) are clearly observed. The peaks at 697 cm^−1^ and 757 cm^−1^ are assigned to the out-of-plane C-H bending vibration of the monosubstituted benzene rings [42], while the band at 1452 cm^−1^ corresponds to the skeleton vibration of the aromatic rings [43]. With increasing CA proportion, the intensities of these PS-associated peaks gradually weaken. At CA/PS = 23/1, the peaks at 757 cm^−1^ and 1452 cm^−1^ almost disappear, and the peak at 697 cm^−1^ is also remarkably attenuated, indicating a substantial decrease in relative PS content within the blend.

In contrast, the characteristic peaks of CA become more pronounced with the elevation of CA content. The band at 1741 cm^−1^ is assigned to the stretching vibration of the ester C=O in acetyl groups of CA. The peak at 1369 cm^−1^ originates from the -CH_3_ bending vibration of acetyl groups, the band at 1231 cm^−1^ corresponds to the ester C-O stretching vibration, and the peak at 1042 cm^−1^ is attributed to the C-O-C stretching of glycosidic bonds on the cellulose molecular backbone [44,45]. Notably, no new peaks emerge, with all peak positions remaining essentially consistent. These results confirm the coexistence of both CA and PS within the electrospun blend nanofibrous membranes and indicate that no chemical reactions occur during the electrospinning process [33].

### 3.3. Thermal Stability Analysis

**Figure 4:**
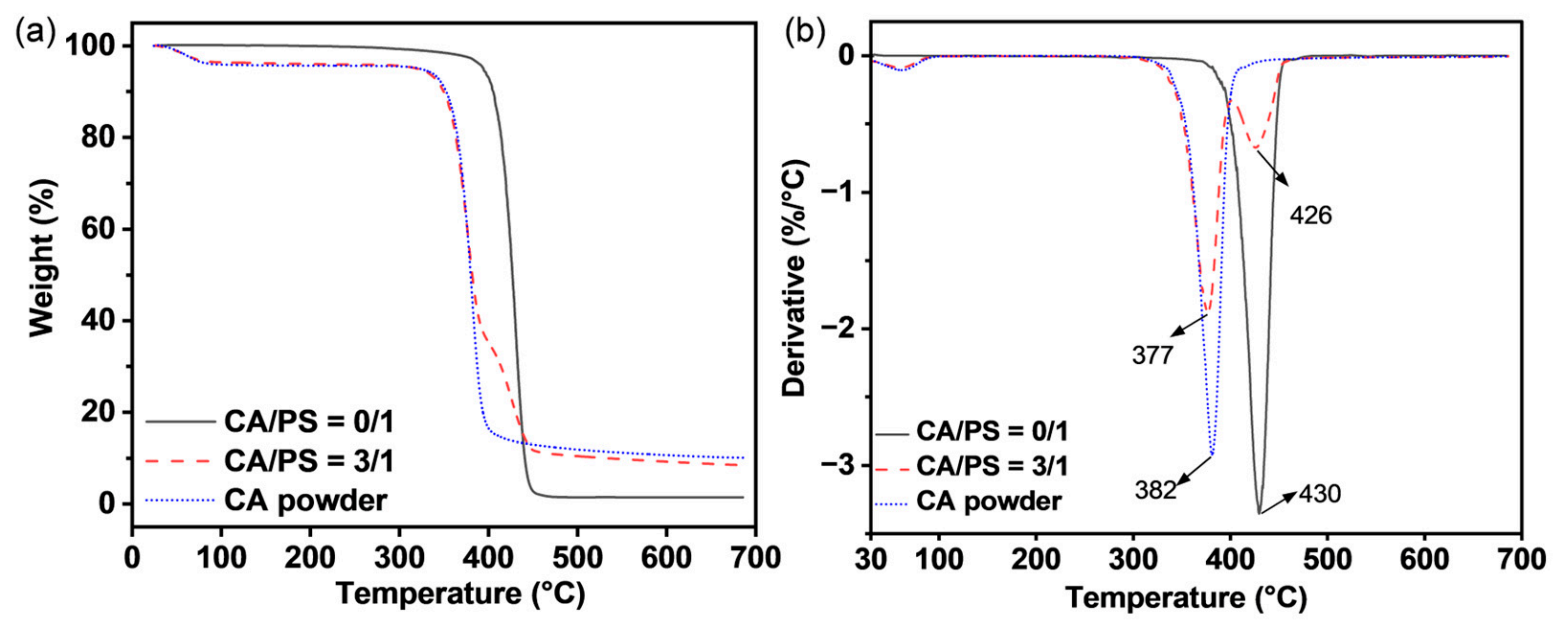
TG (**a**) and DTG (**b**) curves of CA/PS (0/1), CA/PS (3/1) nanofibrous membranes, and pure CA powder.

Figure 4 displays the TG and first derivative of the TG curves of the pure PS membrane (CA/PS = 0/1), CA/PS (3/1) composite membrane, and pure CA powder. The degradation parameters are summarized in Table S2. The pure PS membrane exhibits the highest thermal stability, with T_5%_ and T_90%_ reaching 394 °C and 441 °C, respectively, due to the fracture of the polystyrene backbone [46]; only 1.48% residual mass remains at 650 °C. In contrast, pure CA powder features a lower onset degradation temperature but a markedly higher residual mass (10.31% at 650 °C), which stems from the oxygen-rich molecular structure of CA. The CA/PS (3/1) blend exhibits degradation temperatures (T_5%_ = 320 °C, T_90%_ = 382 °C) comparable to those of pure CA powder. The DTG curve of the CA/PS (3/1) nanofibrous membranes displays two distinct peaks at 377 °C and 426 °C, with almost no overlap between them, corresponding to the maximum decomposition rates of CA and PS, respectively. This suggests that CA and PS retain their independent thermal decomposition behavior within the blend fibers.

### 3.4. Tensile Properties

**Figure 5:**
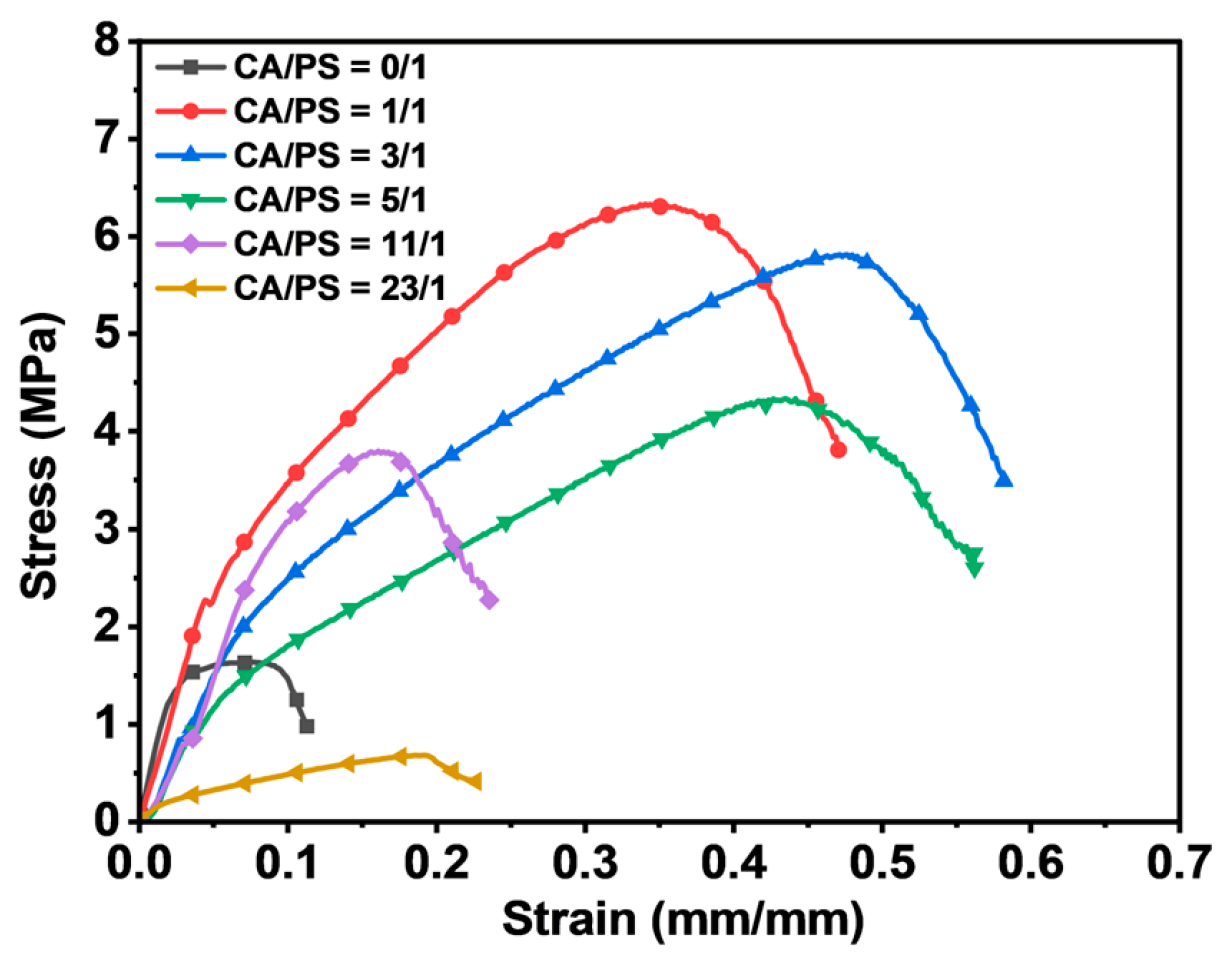
Stress–strain curves of CA/PS nanofibrous membranes with different CA/PS mass ratios.

Mechanical performance is one of the most important evaluation indicators for the practical application of electrospun fibers. However, the mechanical properties of CA/PS blend membranes remain rarely studied (detailed comparison in Table S3).The stress–strain curves of CA/PS membranes with varying blending ratios are displayed in Figure 5, and the corresponding breaking stress and strain values are listed in Table 1. The pure PS membrane (CA/PS = 0/1) exhibits a low breaking stress of 1.50 ± 0.27 MPa and a limited elongation at break of 7.67 ± 1.61%, showing pronounced brittle fracture behavior. Compared with the pure PS membrane, the incorporation of CA significantly improved the elongation at break (*p* < 0.05). On the one hand, the surface grooves and wrinkled textures formed on the blend fibers enhance mechanical interlocking between adjacent fibers, facilitating efficient stress transfer across the fibrous network [47]. On the other hand, the CA/PS (3/1) membrane possesses the maximum fiber diameter; thicker fibers can accommodate greater local deformation and delay crack propagation under tensile loading. Consequently, this material achieves the highest elongation at break (47.12 ± 3.28%). However, excessive CA addition (CA/PS = 23/1) leads to a sharp decline in strength, which is highly consistent with the severe bead-on-string microstructure observed in the SEM results [48].

### 3.5. Porosity

**Figure 6:**
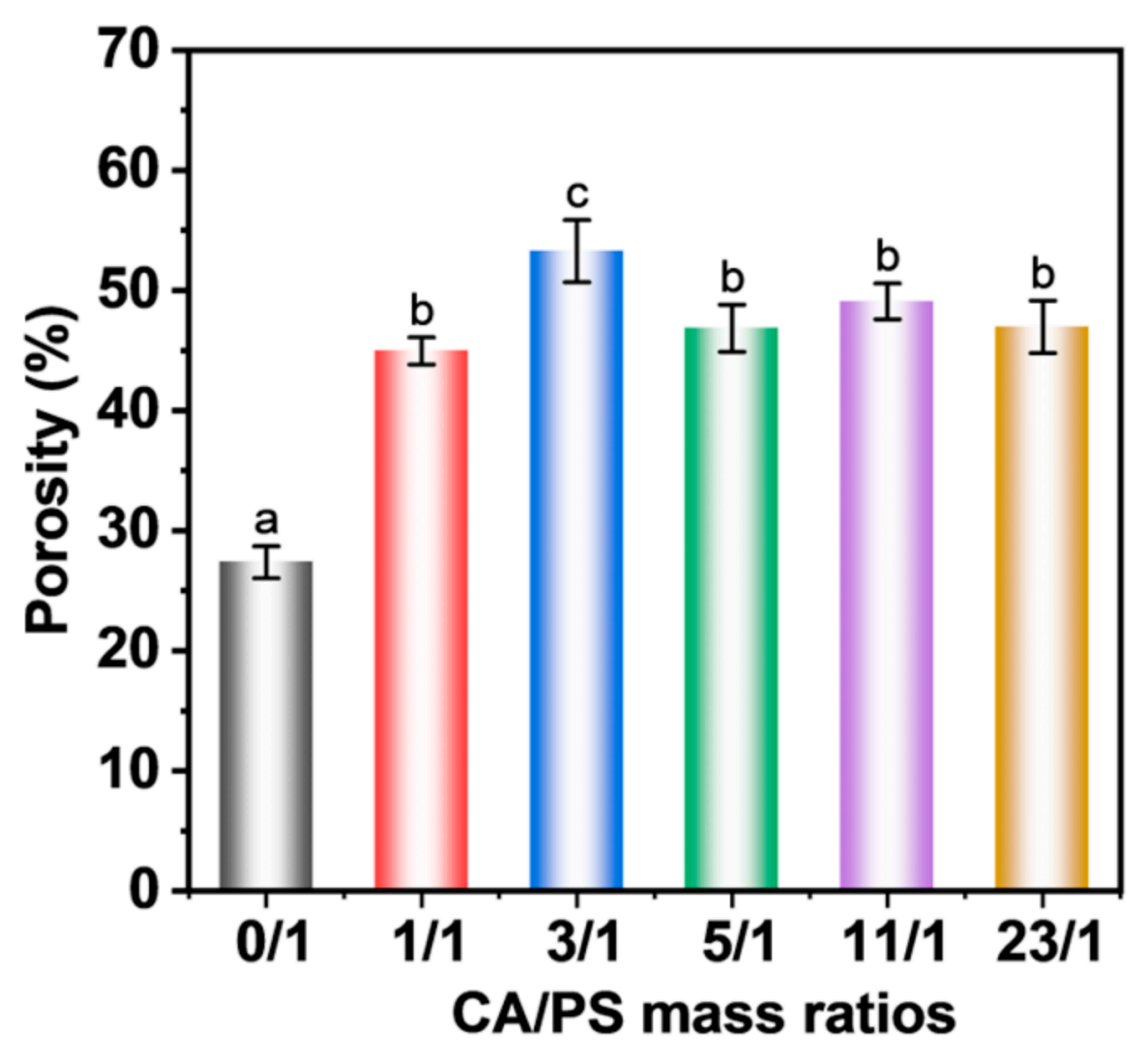
Porosity of CA/PS nanofibrous membranes with different CA/PS mass ratios. Data are expressed as mean ± SD (n = 6). Different uppercase letters above each bar denote significant differences (*p* < 0.05).

Porosity directly characterizes the microstructural features and determines the functional performance of electrospun nanofibrous membranes. The porosity values of CA/PS nanofibrous membranes with different mass ratios are presented in Figure 6. The pure PS membrane (CA/PS = 0/1) exhibits the lowest porosity of 27.38%, which can be attributed to the dense packing of smooth, uniform PS fibers. With the introduction of CA, the porosity significantly increases to 44.97% at CA/PS = 1/1, and reaches the maximum value of 53.27% at CA/PS = 3/1. This enhancement is likely associated with the increased surface roughness and grooved fiber topography, which create additional internal voids within the fibrous network. When the CA proportion further increases to 5/1 or higher, the porosity decreases compared to that of CA/PS = 3/1 (*p* < 0.05). This may be explained by the reduced fiber diameters and more compact fiber arrangement at elevated CA proportions (Figure S4) [49], partially offsetting the pore-forming effect and thus lowering the overall porosity of composite membranes.

### 3.6. Surface Wettability and Waterproofing Performance

**Figure 7:**
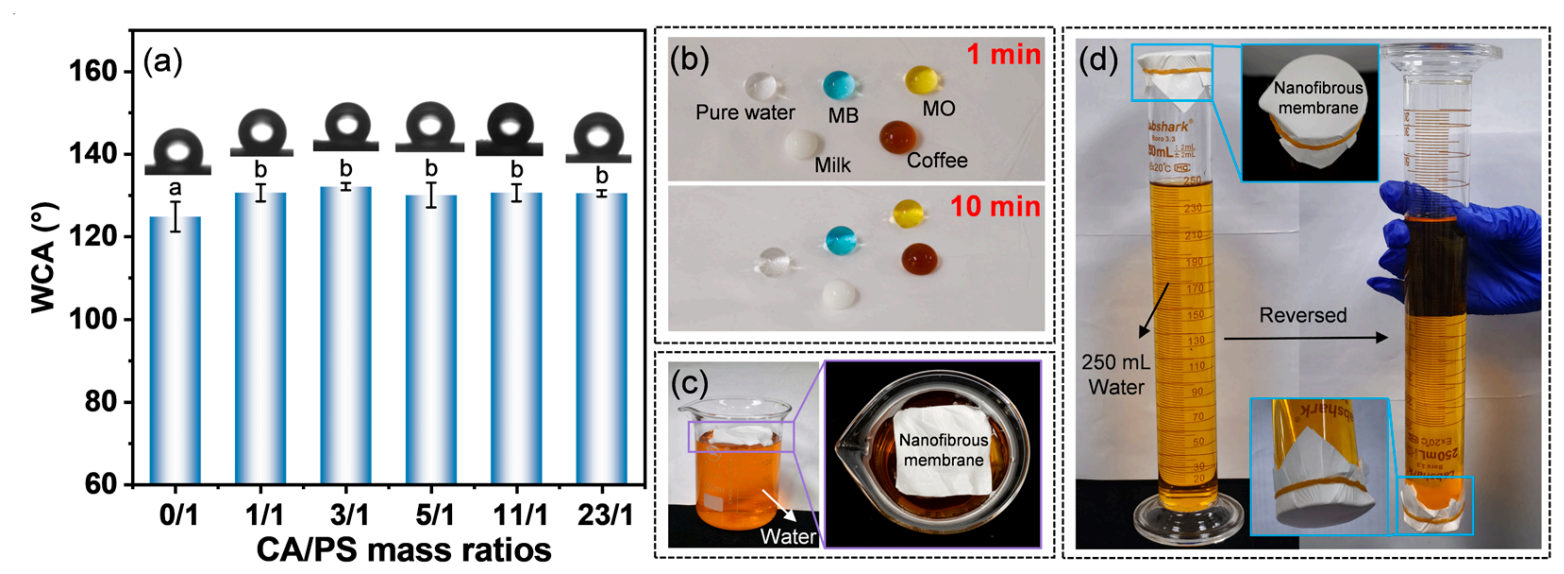
Surface wettability and waterproof performance of CA/PS nanofibrous membranes at various CA/PS mass ratios. (**a**) WCA values at different CA/PS ratios; data are presented as mean ± SD (n = 5). Different letters denote significant differences (*p* < 0.05). (**b**–**d**) Demonstrations using the CA/PS = 3/1 membrane: (**b**) photographs of various liquids resting on the membrane surface after 1 min and 10 min; (**c**) photographs of the membrane floating on water; (**d**) inverted-cylinder test for waterproof performance.

Figure 7 presents the surface wettability of the CA/PS fibrous membranes with different blend ratios, together with a visual hydrophobic demonstration of the representative sample (CA/PS = 3/1). The pure PS membrane exhibits a WCA of 124.79 ± 3.21°, while all blend membranes show significantly higher values (*p* < 0.05). No significant differences in WCA are observed among different CA/PS mass ratios (*p* > 0.05), indicating that the CA/PS ratio within the investigated range has a negligible effect on surface wettability.

The liquid-repellent property of the CA/PS nanofibrous membrane was further evaluated by placing five kinds of liquids (pure water, MB-stained water, MO-stained water, milk, and coffee) on the membrane surface (Figure 7b). All droplets maintained near-spherical shapes without wetting or penetration over the 10 min observation period. Owing to its low weight and hydrophobicity, the membrane floats on the water surface without wetting or sinking (Figure 7c). In addition, an inverted-cylinder test using 250 mL of dyed water sealed by the membrane displays no liquid leakage (Figure 7d), qualitatively indicating the membrane’s waterproof capability.

### 3.7. Breathability of Air and Water Vapor

**Figure 8:**
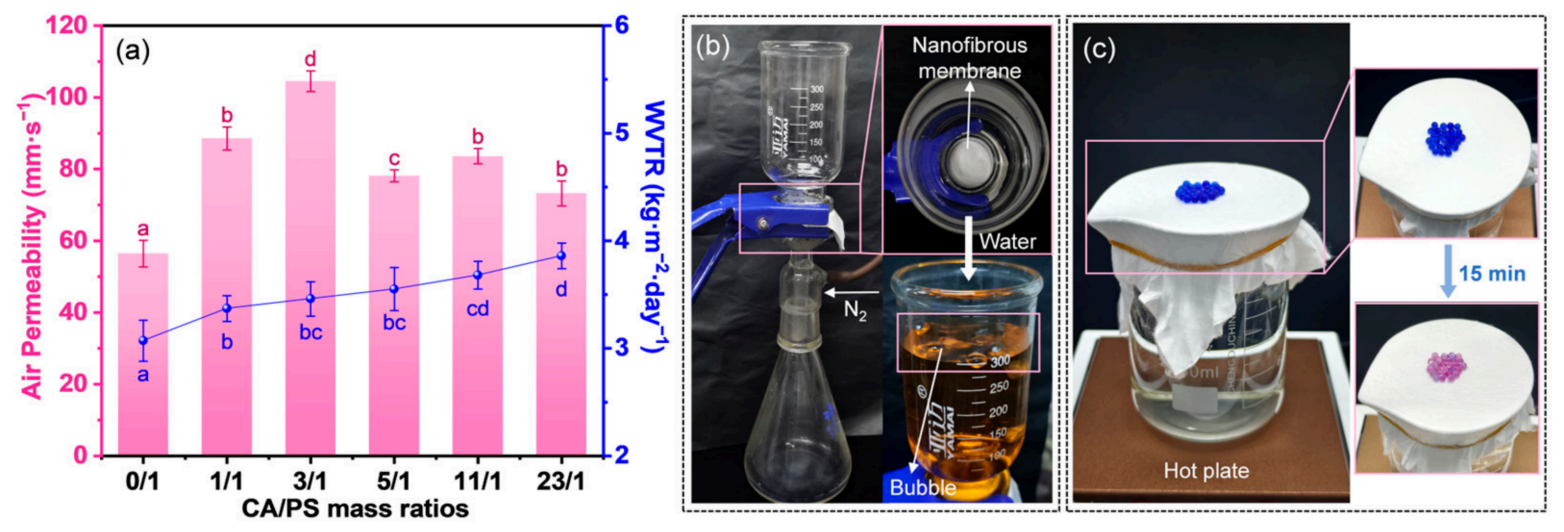
Air permeability and water vapor transmission performance of CA/PS nanofibrous membranes with different CA/PS mass ratios. (**a**) Air permeability and WVTR values; data are presented as mean ± SD (n = 6). Different letters denote significant differences between the samples (*p*  <  0.05). (**b**,**c**) Demonstrations using the CA/PS = 3/1 membrane: (**b**) gas permeability and (**c**) moisture permeability.

The air permeability and WVTR of the CA/PS membranes at different mass ratios are illustrated in Figure 8. Notably, the air permeability follows a variation trend consistent with that of the porosity. The pure PS membrane delivers the lowest air permeability (56.45 ± 3.71 mm/s). The air permeability reaches a maximum of 104.51 ± 2.89 mm/s at the CA/PS mass ratio of 3/1, which can be ascribed to the increased porosity (from 27.38% to 53.27%). In contrast, the WVTR exhibits a distinct tendency. The pure PS membrane (CA/PS = 0/1) produces the lowest WVTR (3.07 ± 0.19 kg·m^−2^·day^−1^), owing to the nonpolar and hydrophobic nature of the PS molecular chains that hinder the adsorption and diffusion of water vapor molecules [50]. Upon the introduction of CA, the WVTR increases gradually, which is attributed to the introduction of polar groups from CA that facilitate water vapor adsorption and diffusion through the membrane.

The qualitative tests in Figure 8b,c further verify the air and moisture permeability of the CA/PS (3/1) nanofibrous membranes. When nitrogen flows through the membrane, gas penetrates the fibrous network and generates bubbles in the dyed water, demonstrating the favorable breathability of the membrane. For moisture permeability evaluation, the membrane was used to seal the opening of a water-filled beaker, with blue silica gel placed on its upper surface. The beaker was then placed on a heating plate at 80 °C. After 15 min, the silica gel changed from blue to pink, which further proves that water vapor can readily pass through the composite nanofibrous membrane.

### 3.8. Infrared Thermal Imaging Analysis

**Figure 9:**
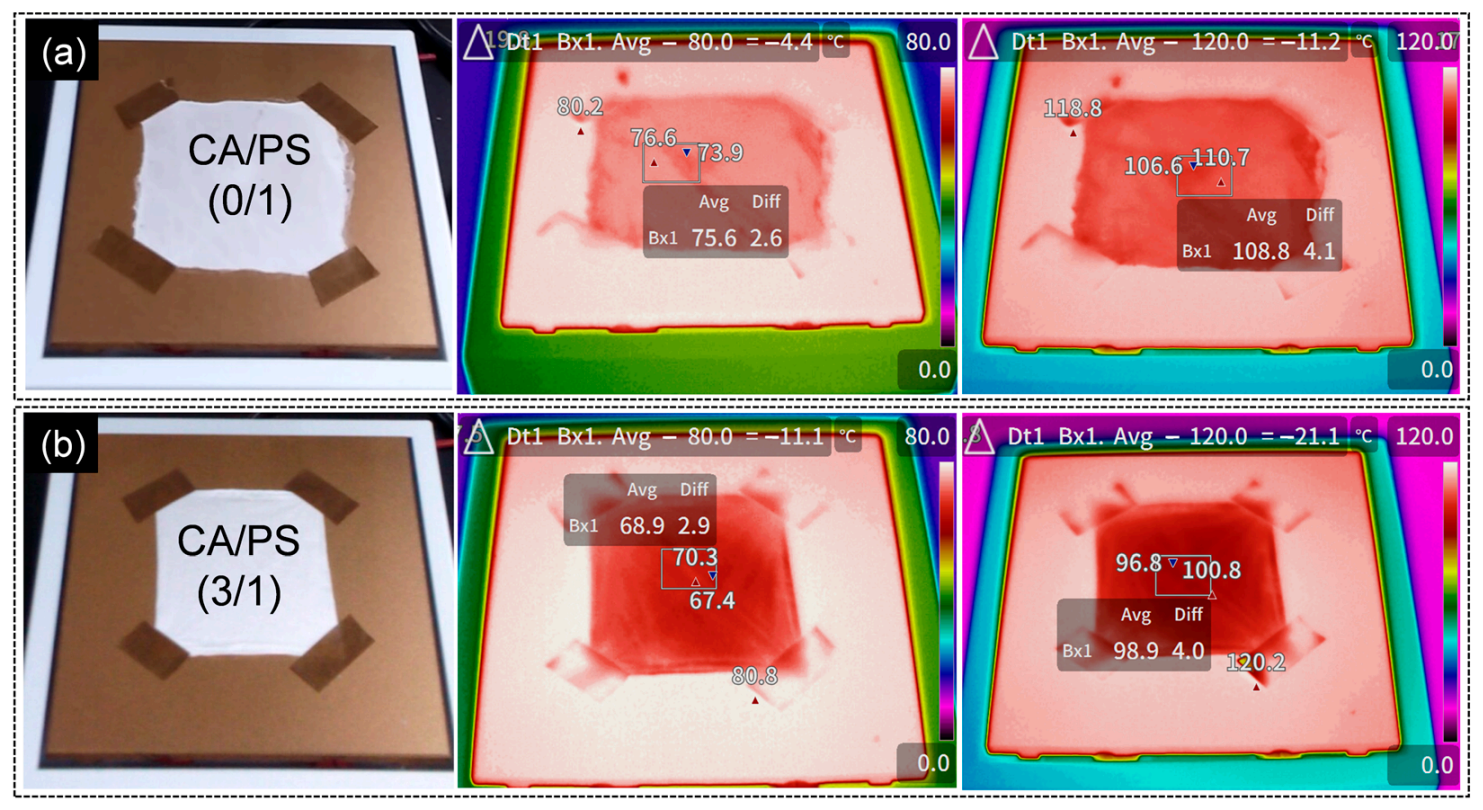
Infrared thermal images of CA/PS (0/1) (**a**) and CA/PS (3/1) (**b**) nanofibrous membranes at platform temperatures of 80 °C and 120 °C. The red and blue triangles are software-generated icons indicating the maximum and minimum temperature points within the tracked area, respectively.

The thermal insulation properties of the pure PS and CA/PS (3/1) nanofibrous membranes were visually assessed by infrared thermal imaging (Figure 9). Under identical heating conditions, the CA/PS (3/1) membrane maintains a distinctly lower surface temperature than the pure PS membrane. Specifically, at platform temperatures of 80 °C and 120 °C, the surface temperatures of the pure PS membrane reached 75.6 °C and 108.8 °C, respectively, whereas those of the CA/PS (3/1) membrane were substantially lower at 68.9 °C and 98.9 °C. The superior thermal insulation of the CA/PS membrane benefits from its highly porous structure; abundant trapped air voids inside the fibrous network effectively suppress heat conduction from the heat source to the membrane surface.

## 4. Conclusions

In this study, CA/PS nanofibrous membranes with tunable fiber diameter, pore architecture, and mechanical properties were successfully fabricated via one-step electrospinning. Adjustment of the CA/PS mass ratio enabled modulation of these structural and mechanical characteristics. The CA/PS (3/1) membrane delivered the optimal overall performance, with a maximum porosity of 53.27%, air permeability of 104.51 ± 2.89 mm/s, and water contact angle of 132.09 ± 0.86°. It achieved well-balanced mechanical properties (breaking stress of 5.38 ± 0.47 MPa and elongation at break of 47.12 ± 3.28%) and enhanced thermal insulation capability compared to pure PS nanofibrous membranes. These results demonstrate the integration of air permeability, mechanical flexibility, and thermal resistance within a single fibrous membrane. Nevertheless, although the membranes exhibit promising comprehensive properties, their practical applications require further validation of application-oriented metrics, including filtration efficiency, oil–water separation capability, and long-term durability. In addition, despite the effectiveness of the THF/DMF solvent system, greener solvent alternatives and residual solvent evaluation are needed to support sustainable scale-up production.

## Figures and Tables

**Table 1:** Breaking stress and elongation at break of CA/PS nanofibrous membranes with different CA/PS mass ratios.

CA/PS Mass Ratios	Breaking Stress (MPa)	Elongation at Break (%)
0/1	1.50 ± 0.27 ^b^ *	7.67 ± 1.61 ^a^
1/1	6.71 ± 0.74 ^e^	34.70 ± 2.56 ^c^
3/1	5.38 ± 0.47 ^d^	47.12 ± 3.28 ^d^
5/1	4.34 ± 1.03 ^cd^	40.09 ± 4.07 ^c^
11/1	3.94 ± 0.38 ^c^	15.60 ± 2.82 ^b^
23/1	0.44 ± 0.31 ^a^	18.38 ± 1.22 ^b^

* Data are expressed as mean ± SD (n = 5). Values marked with different superscript letters within the same column are significantly different (*p* < 0.05).

## Data Availability

The original contributions presented in this study are included in the article/Supplementary Material. Further inquiries can be directed to the corresponding author.

## Supplementary Materials

The following supporting information can be downloaded at: https://www.mdpi.com/article/10.3390/ma19184000/s1, Figure S1: Solution viscosity and electrical conductivity of CA/PS spinning solutions with different mass ratios; Figure S2: SEM image of pure CA electrospun at 14 wt% in THF/DMF (2:1, *w*/*w*); Figure S3: Photographs of spinning solutions with different CA/PS mass ratios; Figure S4: Density and thickness of CA/PS nanofibrous membranes with different CA/PS mass ratios; Table S1: Electrospinning parameters for CA/PS nanofibrous membranes; Table S2: Thermal properties of pure PS (0/1), CA/PS (3/1) nanofibrous membranes, and pure CA powder; Table S3: Comparison of key properties of electrospun CA/PS nanofibrous membranes between the present work and previous studies.

## References

[1] Ding M., Wang Y., Gong X., Luo M., Yin X., Yu J., Zhang S., Ding B. (2025). Fluorine-Free Nanofiber/Network Membranes with Interconnected Tortuous Channels for High-Performance Liquid-Repellency and Breathability. ACS Nano.

[2] Wu H., Shen X., Wei M., Zhang F., Low Z.-X., Xing W. (2026). Engineering electrospun hydrophobic SiO2 nanofibers via organosilica-based click chemistry modification for ultrafast oil-water separation. J. Membr. Sci..

[3] Wu Y., Jin M., Zhai Z., Li Y., Yang B. (2026). Experimental study on a superhydrophobic and superoleophilic nanofiber membrane with vine-interwoven hierarchical structures for capturing performance of oily particulate filtration. Powder Technol..

[4] Su W., Chang Z., E Y., Feng Y., Yao X., Wang M., Ju Y., Wang K., Jiang J., Li P. (2024). Electrospinning and electrospun polysaccharide-based nanofiber membranes: A review. Int. J. Biol. Macromol..

[5] El-Samak A.A., Ponnamma D., Hassan M.K., Adham S., Karim A., Ammar A., Alser M., Shurbaji S., Eltai N.O., Al-Maadeed M.A.A. (2021). Multifunctional Oil Absorption with Macroporous Polystyrene Fibers Incorporating Silver-Doped ZnO. ACS Omega.

[6] Feng T., Fu L., Mu Z., Wei W., Li W., Liang X., Ma L., Wu Y., Wang X., Wu T. (2025). Bicomponent Electrospinning of PVDF-Based Nanofiber Membranes for Air Filtration and Oil–Water Separation. Polymers.

[7] Sadeghi-Ghadikolaei M., Vasheghani-Farahani E., Bagheri F., Khorrami Moghaddam A., Mellati A., Karimizade A. (2024). Fabrication of 3D chitosan/polyvinyl alcohol/brushite nanofibrous scaffold for bone tissue engineering by electrospinning using a novel falling film collector. Int. J. Biol. Macromol..

[8] Shen M., Liu H., Pan T., Ning J., Zhou D., Song G., Wang Y., Cai S., Xia X., Zhang G. (2023). Crosslinked PVA electrospinning nanofibrous film as a new platform for the design of K+ sensor. Sens. Actuators B. Chem..

[9] Gong X., Yin X., Wang F., Liu X., Yu J., Zhang S., Ding B. (2023). Electrospun Nanofibrous Membranes: A Versatile Medium for Waterproof and Breathable Application. Small.

[10] Omiyale B.O., Ogbeyemi A., Rasheed A.A., Adamolekun T.M., Zhang W.C. (2025). Influence of electrospinning parameters on the development of high-quality electrospun nanofibers: A brief critical assessment. Next Nanotechnol..

[11] Han Y., Xu Y., Zhang S., Li T., Ramakrishna S., Liu Y. (2020). Progress of Improving Mechanical Strength of Electrospun Nanofibrous Membranes. Macromol. Mater. Eng..

[12] Ma S., Li A., Pan L. (2024). Application Progress of Multi-Functional Polymer Composite Nanofibers Based on Electrospinning: A Brief Review. Polymers.

[13] Wu Y., Liu C., Yu J., Cho Y., Baek J.W., Kim I.-D., Ding B. (2026). Single Nanofiber Mechanics: Guiding Principles for Robust and Multifunctional Nanofiber Materials. ACS Nano.

[14] Chinnappan B.A., Krishnaswamy M., Xu H., Hoque M.E. (2022). Electrospinning of Biomedical Nanofibers/Nanomembranes: Effects of Process Parameters. Polymers.

[15] Hami S.S.B.M., Affandi N.D.N., Indrie L., Tripa S., Harun A.M., Ahmad M.R. (2023). Enhancing Mechanical Properties and Flux of Nanofibre Membranes for Water Filtration. Polymers.

[16] Nauman S., Lubineau G., Alharbi H.F. (2021). Post Processing Strategies for the Enhancement of Mechanical Properties of ENMs (Electrospun Nanofibrous Membranes): A Review. Membranes.

[17] Cai J., Zhang Q., Lei M., He J., Liu G. (2016). The use of solvent-soaking treatment to enhance the anisotropic mechanical properties of electrospun nanofiber membranes for water filtration. RSC Adv..

[18] Zheng Z., Zhang H., Yang J., Liu X., Chen L., Li W., Mi S., Zhou H., Zheng W., Xue W. (2025). Recent advances in structural and functional design of electrospun nanofibers for wound healing. J. Mat. Chem. B.

[19] Akhila B., Abhijith V., Sreedharan M., Ravindran L., Sathian A., Thomas S., Sadasivan S.M. (2025). Innovations in Core–Shell Electrospinning: A Comprehensive Review in Recent Advances of Core–Shell Electrospun Polylactic Acid Nanocomposite Fibers for Potential Biomedical Applications. ACS Biomater. Sci. Eng..

[20] Yang T., Zhang W., Giri S., Thakur R., Sarkar P., Khan A., Riahi Z., Assadpour E., Zhang W., Jafari S.M. (2026). Fibers/nanofibers from hybrid biopolymeric systems: Tailoring blending strategies for enhanced electrospinning in sustainable food packaging. Adv. Colloid Interface Sci..

[21] Adeli B., Gharehaghaji A.A., Jeddi A.A.A. (2023). Energy Harvesting by Cyclic Tensile Loading and Buckling via an Electrospun Polyblend Elastic Layer of PVDF/PU. Fibers Polym..

[22] Weng Q.-H., Hu M.-H., Wang J.-F., Hu J.-J. (2025). Enhancing the Flexibility and Hydrophilicity of PLA via Polymer Blends: Electrospinning vs. Solvent Casting. Polymers.

[23] Wang K., Guo J., Wang T., Yuan H., Gao Y., Cui X. (2025). Advances in polystyrene-based oil-absorbent materials: A review. J. Environ. Chem. Eng..

[24] Niknejad A.S., Bazgir S., Kargari A. (2021). Mechanically improved superhydrophobic nanofibrous polystyrene/high-impact polystyrene membranes for promising membrane distillation application. J. Appl. Polym. Sci..

[25] Akanbi M.J., Jayasinghe S.N., Wojcik A. (2021). Characterisation of electrospun PS/PU polymer blend fibre mat for oil sorption. Polymer.

[26] Li P., Qiao Y., Zhao L., Yao D., Sun H., Hou Y., Li S., Li Q. (2015). Electrospun PS/PAN fibers with improved mechanical property for removal of oil from water. Mar. Pollut. Bull..

[27] Jiang Z., Tijing L.D., Amarjargal A., Park C.H., An K.-J., Shon H.K., Kim C.S. (2015). Removal of oil from water using magnetic bicomponent composite nanofibers fabricated by electrospinning. Compos. B Eng..

[28] Yoon J.W., Park Y., Kim J., Park C.H. (2017). Multi-jet electrospinning of polystyrene/polyamide 6 blend: Thermal and mechanical properties. Fash. Text..

[29] Gupta P., Mishra S., Ullas A.V. (2025). Electrospun polystyrene fibres: Influence of polymer concentration on the oil sorption behaviour. Polym. Bull..

[30] Ji S.-H., Yun J.-S. (2022). Highly Porous-Cellulose-Acetate-Nanofiber Filters Fabricated by Nonsolvent-Induced Phase Separation during Electrospinning for PM_2.5_ Capture. Nanomaterials.

[31] Oldal D.G., Topuz F., Holtzl T., Szekely G. (2023). Green Electrospinning of Biodegradable Cellulose Acetate Nanofibrous Membranes with Tunable Porosity. ACS Sustain. Chem. Eng..

[32] Rosdi N., Mohd Kanafi N., Abdul Rahman N. (2018). Preparation and Thermal Properties of Cellulose Acetate/Polystyrene Blend Nanofibers via Electrospinning Technique. Pertanika J. Sci. Technol..

[33] Abosedira S., Soliman M., Ebrahim S., Fadl E., Khalil M. (2025). A practical strategy of electrospun fibers of polystyrene/cellulose acetate blend for atmospheric water harvesting. Alex. Eng. J..

[34] Wu X.-W., Seenivasan M., Karuppiah C., Zhang B.-R., Shih J.-Y., James Li Y.-J., Hung T.-F., Chien W.-C., Ramaraj S.K., Jose R. (2024). Fabrication electro-spun Poly(vinyl alcohol)-Melamine nonwoven membrane composite separator for high-power lithium-ion batteries. Heliyon.

[35] (2016). Standard Test Methods for Water Vapor Transmission of Materials.

[36] Bertolo-Cagnoto M.R.V., Alvarenga A.D., de Camargo L.A., Costa K.B.T., Correa D.S. (2026). Natural deep eutectic solvent-assisted incorporation of anthocyanins into cellulose acetate nanofibers for halochromic sensors. Int. J. Biol. Macromol..

[37] Nuamcharoen P., Kobayashi T., Potiyaraj P. (2021). Influence of volatile solvents and mixing ratios of binary solvent systems on morphology and performance of electrospun poly(vinylidene fluoride) nanofibers. Polym. Int..

[38] Zaarour B., Zhang W.X., Zhu L., Jin X.Y., Huang C. (2019). Maneuvering surface structures of polyvinylidene fluoride nanofibers by controlling solvent systems and polymer concentration. Text. Res. J..

[39] Silva G.A., Eckelt J., Gonçalves M.C., Wolf B.A. (2003). Thermodynamics of pseudo-ternary systems as a tool to predict the morphologies of cellulose acetate/polystyrene blends cast from tetrahydrofuran solutions. Polymer.

[40] Wang L.F., Pai C.L., Boyce M.C., Rutledge G.C. (2009). Wrinkled surface topographies of electrospun polymer fibers. Appl. Phys. Lett..

[41] Liu W., Huang C., Jin X. (2015). Electrospinning of Grooved Polystyrene Fibers: Effect of Solvent Systems. Nanoscale Res. Lett..

[42] Meng T.K., Kassim A.S.B.M., Razak A.H.B., Fauzi N.A.B.M. (2021). *Bacillus megaterium*: A Potential and an Efficient Bio-Degrader of Polystyrene. Braz. Arch. Biol. Technol..

[43] Abdelhafiz M., Hussein A.K., Khalil W.F., Elbeih A. (2023). An energetic nano-fiber composite based on polystyrene and 1,3,5-trinitro-1,3,5-triazinane fabricated via electrospinning technique. Def. Technol..

[44] Wilkinson E., Hoh E., Stack M., Mladenov N., Youssef G. (2025). Thermal, Physical, Chemical, and Mechanical Properties of Cellulose Acetate From Cigarette Filters. J. Appl. Polym. Sci..

[45] Yu X., Zhang X., Xing Y., Zhang H., Jiang W., Zhou K., Li Y. (2021). Development of Janus Cellulose Acetate Fiber (CA) Membranes for Highly Efficient Oil–Water Separation. Materials.

[46] Vanapalli K.R., Bhattacharya J., Samal B., Chandra S., Medha I., Dubey B.K. (2021). Inhibitory and synergistic effects on thermal behaviour and char characteristics during the co-pyrolysis of biomass and single-use plastics. Energy.

[47] Geng R., Wang Y., Su Z., Li H., Li H., Song Z. (2026). Wrinkled SiBCN ceramic aerogels fabricated by microfluidic electrospinning for shrinkage resistance and thermal insulation. Ceram. Int..

[48] Abdullah M.F., Andriyana A., Muhamad F., Ang B.C. (2021). Effect of core-to-shell flowrate ratio on morphology, crystallinity, mechanical properties and wettability of poly(lactic acid) fibers prepared via modified coaxial electrospinning. Polymer.

[49] Wang N., Burugapalli K., Song W., Halls J., Moussy F., Zheng Y., Ma Y., Wu Z., Li K. (2013). Tailored fibro-porous structure of electrospun polyurethane membranes, their size-dependent properties and trans-membrane glucose diffusion. J. Membr. Sci..

[50] Röhrl M., Ködel J.F., Timmins R.L., Callsen C., Aksit M., Fink M.F., Seibt S., Weidinger A., Battagliarin G., Ruckdäschel H. (2023). New Functional Polymer Materials via Click Chemistry-Based Modification of Cellulose Acetate. ACS Omega.

