# Supplementary material for: Preparation and Multifunctional Properties of Hydrophobic Electrospun Cellulose Acetate/Polystyrene Nanofibrous Membranes
Source: Materials (Basel). 2026 Sep 20;19(18):4000. doi: 10.3390/ma19184000 (PMC13608400; doi:10.3390/ma19184000)
Supplement: Supplementary file 1 [file materials-19-04000-s001.zip › materials-4520832-supplementary-final.pdf]

Supporting Information

Preparation and Multifunctional Properties of Hydrophobic Electrospun Cellulose Acetate/Polystyrene Nanofibrous Membranes

Zijia Wang, Fujuan Liu *

National Engineering Laboratory for Modern Silk, College of Textile and Clothing Engineering, Soochow University, 199 Ren-Ai Road, Suzhou 215123, China

* Corresponding author. Email: liufujuan@suda.edu.cn

Table S1. Electrospinning parameters for CA/PS nanofibrous membranes.

Parameters	Values
Applied voltage	18 kV
Tip-to-collector distance	16 cm
Feeding rate	1 mL/h
Needle gauge	21 G (inner diameter 0.51 mm, outer 0.82 mm)
Syringe volume	10 mL
Collector type	Metallic drum collector (diameter 100 mm, length 200 mm)
Collector rotation speed	200 rpm
Deposition time	8 h
Temperature	25 ± 2 ℃
Relative humidity	45 ± 5%
Drying condition	60 ℃ for 6 h

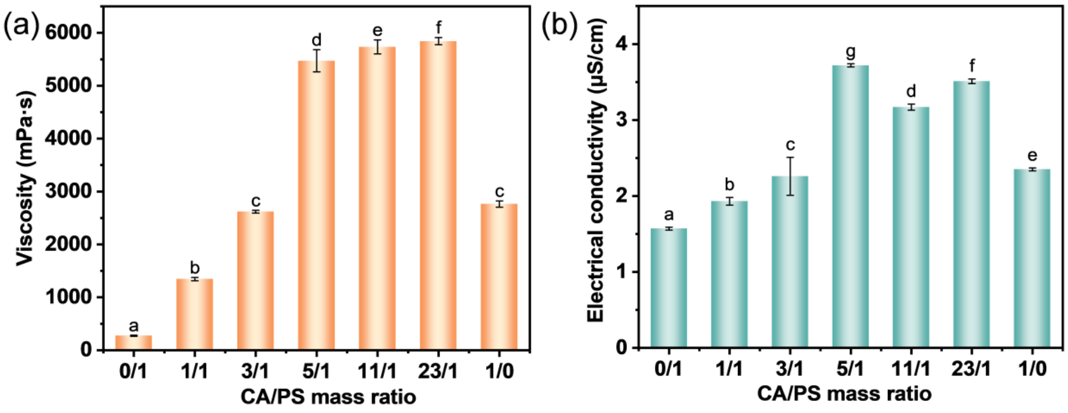

Figure S1. Solution viscosity and electrical conductivity of CA/PS spinning solutions with different mass ratios: (a) viscosity and (b) conductivity. Data are expressed as mean ± SD (n = 3). Different uppercase letters above the bars denote significant differences ($p < 0.05$).

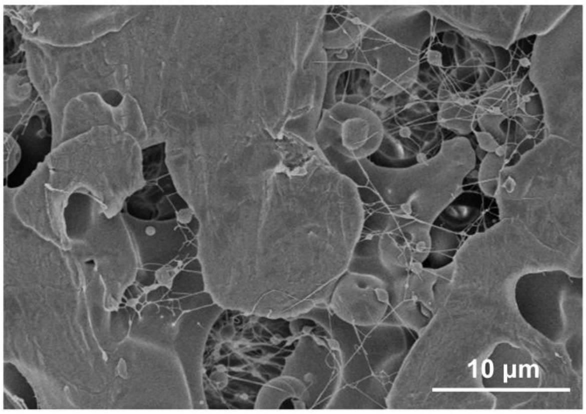

Figure S2. SEM image of pure CA electrospun at 14 wt% in THF/DMF (2:1, w/w) under identical spinning conditions.

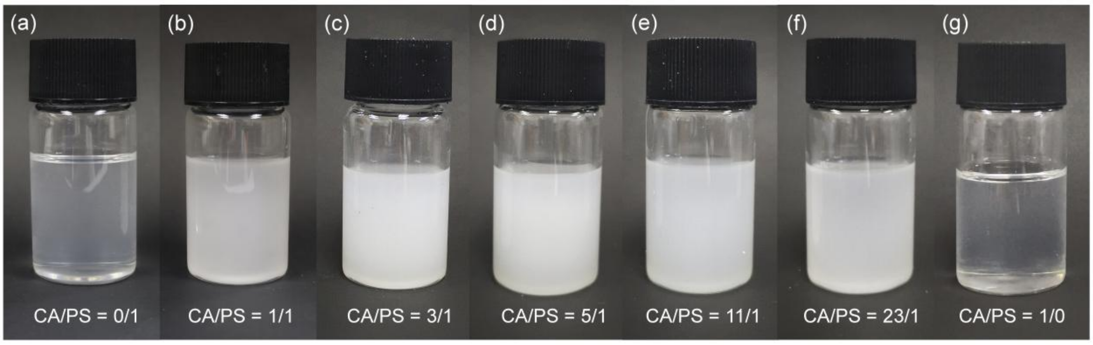

Figure S3. Photographs of spinning solutions with different CA/PS mass ratios: (a) pure PS (0/1), (b) 1/1, (c) 3/1, (d) 5/1, (e) 11/1, (f) 23/1, and (g) pure CA (1/0). All solutions were prepared at a total polymer concentration of 14 wt% in THF/DMF (2:1, w/w).

Table S2. Thermal properties of pure PS (0/1), CA/PS (3/1) nanofibrous membranes, and pure CA powder derived from TGA and DTG curves.

Sample	CA/PS (0/1)	CA/PS (3/1)	Pure CA powder
$T_{5\%}$ (℃) [a]	394	320	323
$T_{10\%}$ (℃) [b]	405	350	353
$T_{50\%}$ (℃) [c]	427	382	380
$T_{90\%}$ (℃) [d]	441	535	-
Residual mass at 200 °C (%)	99.97	96.02	95.67
Residual mass at 650 °C (%)	1.48	8.78	10.31

[a, b, c, d] $T_{5\%}$, $T_{10\%}$, $T_{50\%}$ and $T_{90\%}$ represent the temperatures at which 5%, 10%, 50% and 90% of the initial sample mass are lost, respectively.

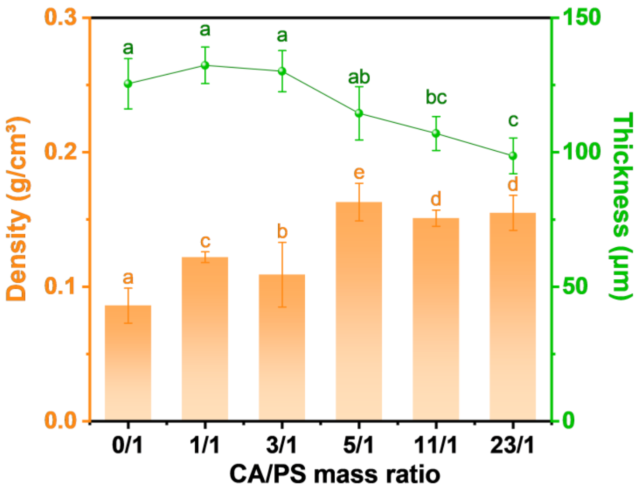

Figure S4. Density and thickness of CA/PS nanofibrous membranes with different CA/PS mass ratios. Data are expressed as mean ± SD (n = 6). Different uppercase letters above the bars denote significant differences ($p < 0.05$).

Table S3. Comparison of key properties of electrospun CA/PS nanofibrous membranes between the present work and previous studies.

Property / Feature	Rosdi et al. (2018) [32]	Abosedira et al. (2025) [33]	This work
CA/PS ratios	20:80, 40:60, 60:40, 80:20, 0:100	0:1, 1:1, 1:2, 1:3, 1:0	0/1, 1/1, 3/1, 5/1, 11/1, 23/1
Main focus	Thermal properties	Water harvesting	Multifunctional properties
Average fiber diameters	100-800 nm	111-249 nm	725-1140 nm
Porosity	-	-	53.27% (CA/PS = 3/1)
Mechanical properties	-	-	Breaking stress: 5.38 ± 0.47 MPa (CA/PS = 3/1)<br>Elongation at break: 47.12 ± 3.28% (3/1)
Wettability (WCA)	-	124.7° (CA:PS = 1:2)	up to 132.09 ± 0.86° (CA/PS = 3/1)
Permeability	-	-	Air permeability: 104.51 ± 2.89 mm/s (CA/PS = 3/1)<br>WVTR: 3.46 ± 0.16 $g \cdot m^{-2} \cdot day^{-1}$ (CA/PS = 3/1)
Thermal properties	TGA and DSC	TGA	TGA
Thermal insulation	-	-	Evaluated (infrared thermal imaging)
